# Free Style Perforator Flaps for Aesthetic Facial Reconstruction

**DOI:** 10.29252/wjps.8.2.195

**Published:** 2019-05

**Authors:** Nader Elmelegy, Sameh Elghamry, Tarek Shoukr

**Affiliations:** 1 Department of Plastic Surgery, Faculty of Medicine, Menoufia University, Menoufia, Egypt; 2 Department of Plastic Surgery, Faculty of Medicine, Tanta University, Tanta, Egypt

**Keywords:** Perforator, Flap, Aesthetic, Facial, Reconstruction

## Abstract

**BACKGROUND:**

Functional and cosmetic outcomes affect reconstruction of the face more than any region of the body. To use a predetermined perforator flap freely designed allowing a wide range of movement and manipulation can give us an optimum outcome. We present our clinical experience with free style facial perforator flaps, surgical technique, and complications.

**METHODS:**

Thirty patients with post-tumor resection of the face were reconstructed with free style local perforator flaps between January 2014 and November 2016. Doppler was used to identify the perforator vessels preoperatively.

**RESULTS:**

Twenty-two clinical cases had no complications. Four had venous congestion that resolved spontaneously, three had a distal 1/3 superficial necrosis, and one suffered from hematoma.

**CONCLUSION:**

Freestyle perforator flaps were applied to get better cosmetic facial reconstruction, allowing one stage procedure and decreasing donor site morbidity. Modern anatomical understanding, good planning, and meticulous surgical technique can affect clinical results.

## INTRODUCTION

Functional and cosmetic outcomes affect reconstruction of the face more than any region of the body. Local facial flaps are an excellent option due to color and texture match of their tissues. The excellent vascularity of facial skin ensures a reliable blood supply to pedicled or islanded local flaps. Limited range of motionand bulkiness at the pedicle site is among the limitations that confront local pedicled flaps and may require secondary surgical revision.^1^ Hofer^2^ was the first to use the free style approach that was introduced by Mardini and Wei in 2004^3^ for facial reconstruction. 

Depending on Doppler signals in a specific region free style perforator flaps can be harvested.^2^^-^^4^ A large arc of rotation which is due to thin pedicle allows these flaps to reach different defects in the face.^5^ The needed primary closure of the harvesting site and pedicle location^6^ can limit the reconstruction by these flaps to small and medium size facial defects.^7^ Gunnarsson and Thomsen (2016) reported a maximum facial perforator flap size of 9×5 cm.^4^

Although Taylor and Palmer in 1987^8^ studied perforator arteries on cadavers and others recently studied perforator vessels in the face,^2^^,^^9^^,^^10^ but clinical studies discussing the use of local perforator flaps for facial reconstruction still not enough, and so clinical applications of these flaps have not been adequately investigated. Surgical advantages of local perforator flaps have been well described,^7^^,^^11^^,^^12^ unlike their cosmetic manipulation or complications. We aimed to present our clinical experience in surgical technique, cosmetic outcome and complications of predetermined free style facial perforator flaps.

## MATERIALS AND METHODS

Thirty patients with facial defects after tumor excision were reconstructed with free style local perforator flaps between January 2014 and November 2016 at the Plastic Surgery Department, Tanta University. Patients ages ranged from 35 to 72 years, 16 were males and 14 were females. Twenty flaps were based on facial artery perforators at the nasolabial region, 7 flaps were based on infraorbital artery perforators and 3 in the post-auricular area. 

Skeletonization of the perforator vessel was done in 12 cases to increase the range of movement, while for the other 18 cases, no skeletonization was done. All the flaps were of the propeller type. The arc of rotation of the propeller flaps ranged from 90 to 180 degrees and 4 of them were pended over to fit into the defect. General anesthesia was used in 20 patients, while the other 10 patients were operated under local anesthesia. Preoperatively, the excision margins and the expected defect were established, then, Doppler was used to identify the perforator vessels around the expected defect. 

**Fig. 1:**
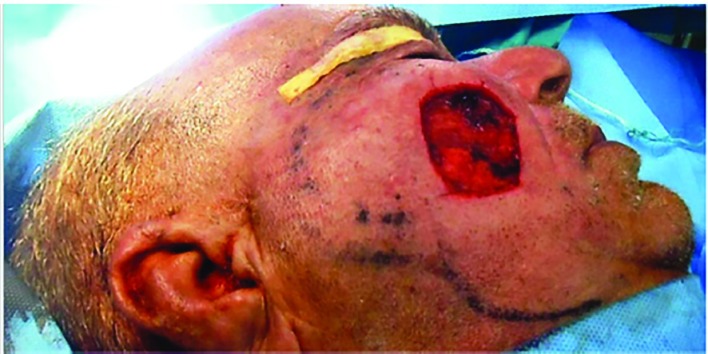
The defect after removing the lesion

**Fig. 2:**
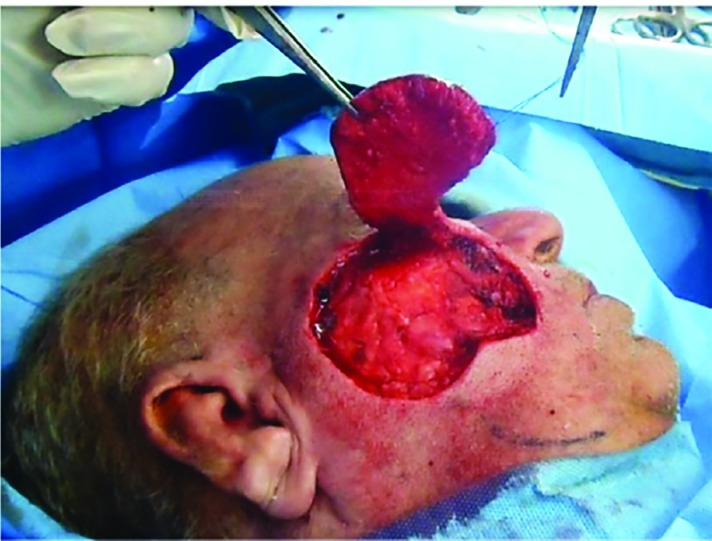
Elevating the flap

**Fig. 3:**
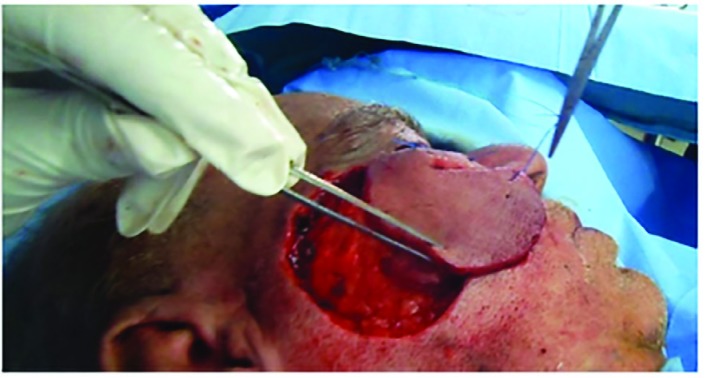
Rotating the flap to cover the defect

**Fig. 4:**
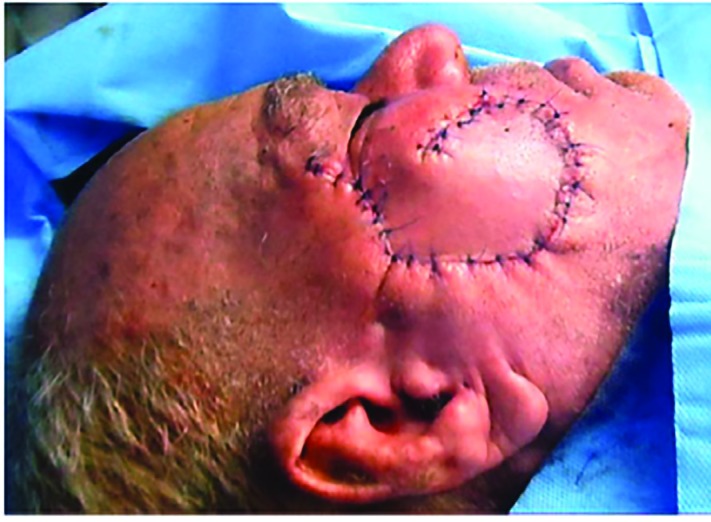
Final flap inset and closure of the defect

One suitable perforator had been chosen and the flap had been designed suitable enough to fit into the expected defect. Intraoperatively, exploration of the chosen perforator had been done before raising the flap to make sure that it was suitable for the vascular supply of the flap and for its proposed movement into the defect. Whether the perforator would be skeletonized or not, would be governed by the needed movement of the flap to be in set without any compromise of its blood supply (Figure 1-4).

## RESULTS

**Fig. 5:**
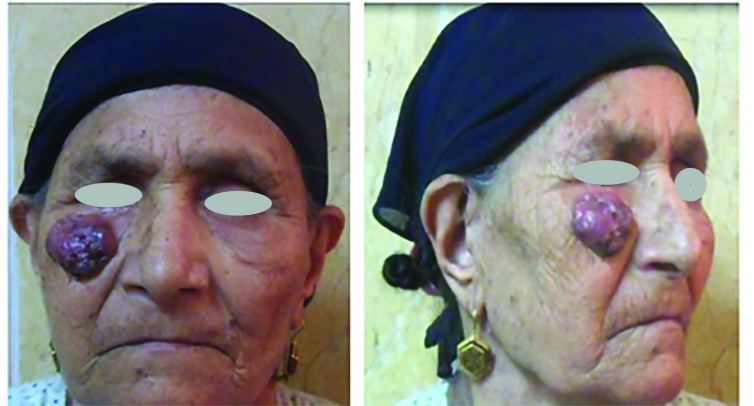
Preoperative front and side views case 1

**Fig. 6:**
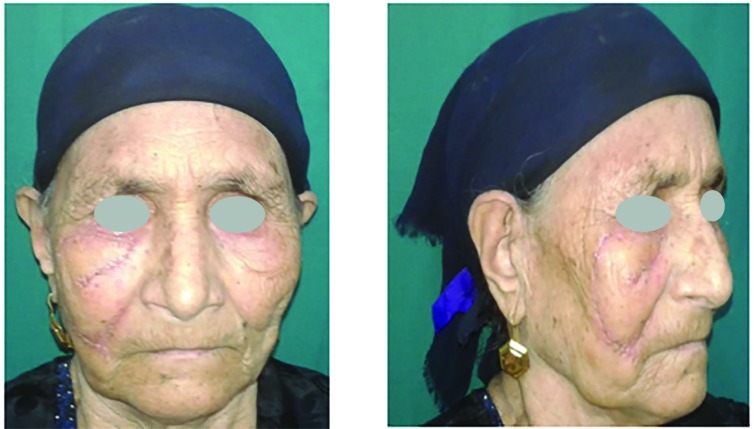
Postoperative front and side views case 1

**Fig. 7:**
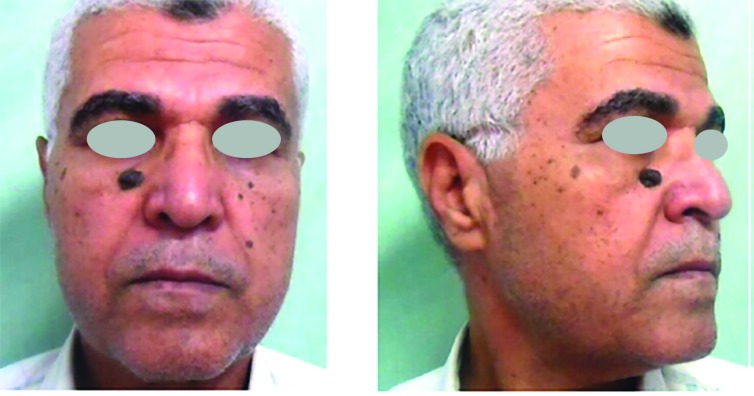
Preoperative front and side views case 2

**Fig. 8:**
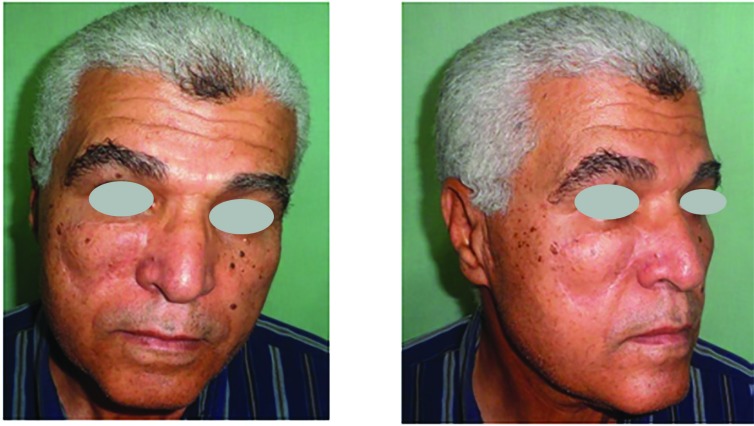
Postoperative front and side views case 2

Histopathological examination of the resected lesions showed 22 basal cell carcinomas, 6 squamous cell carcinomas, and 2 melanomas. No recurrence was observed during the follow-up period. Twenty-two cases had no complications, three had venous congestion that resolved spontaneously within 2 days and they were the three flaps that had been bent over to fit into the malar defect, three had a distal 1/3 superficial necrosis, and 2 suffered hematoma that needed evacuation under local anesthesia (Figure 5-8).

## DISCUSSION

The knowledge of a perforator-based design evolved from the angiosomal concept introduced by Taylor and Palmer in 1987.^8^ Blondeel *et al.* described a perforating vessel as a vessel that had its origin in one of the axial vessels of the body and passed through different structures, perforating the deep fascia before reaching the skin.^13^ Hofer *et al.* in 2005 described facial artery perforator flap and indicated the location of perforators.^2^

D’Arpa *et al.* published a series of nasolabial perforator flaps for alar reconstruction and reported that facial artery perforators pierce the superficial musculoaponeurotic system layer due to absence of deep fascia in the face.^14^ Qassemyar *et al. *reported in 20 cadaver dissections, the perforasomes of the facial artery perforators.^10^ Gunnarson *et al.* published the advantages of using color Doppler ultrasound to localize facial artery perforators and this agreed with our technique in using Doppler identification of perforators in preoperative planning.^4^

On the other hand, other studies suggested that localization of suitable perforator vessels in the face cannot be guaranteed by Doppler due to the anatomical features of these regions.^7^^,^^12^^,^^14^^,^^15^^,^^16^ With experience, we could rely on the use of Doppler for planning perforator flaps of the face although of the superficial position of the axial arteries of the face that can be confused with the perforators. Free style perforator flaps can be based on one or more perforators obtaining a reliable blood supply together with great versatility in design, free choice of orientation, arc of rotation up to 180°, wider rangeof motion compared with local flaps, and primary closure of the donor site along the relaxed skin tension lines to minimize scarring. 

These flaps, based on a perforator from a known axial vessel, can be realized in different areas such as nasolabial sulcus, peri-oral, peri-zygomatic, and submental region.^2^^,^^4^^,^^6^^-^^15^^,^^17^^,^^18^ During dissection, it is necessary to leave a cuff of subcutaneous fatty tissue around the artery to avoid pedicle kinking and to choose a safe sense of rotation in 180° propeller before in setting.^18^ We recommend accurate selection of patients and identification of possible risk factors which can lead to complications such as diabetes, smoking, radiation, and immune-suppression.^19^ In conclusion, free style local perforator flaps are useful for cosmetic reconstruction of complex facial defects because of their versatility, wide arc of rotation, similar texture, and color match with pleasing results.

## CONFLICT OF INTEREST

The authors declare no conflict of interest.
